# Aconselhamento breve em consultas de rotina: uma estratégia populacional para reduzir a carga da doença e econômica do tabagismo no Brasil

**DOI:** 10.1590/0102-311XPT000125

**Published:** 2025-08-08

**Authors:** Andre S. Szklo

**Affiliations:** 1 Coordenação de Prevenção e Vigilância, Instituto Nacional de Câncer, Rio de Janeiro, Brasil.

**Keywords:** Cessação do Tabagismo, Política Pública, Pessoal de Saúde, Smoking Cessation, Public Policy, Health Personnel, Cese del Hábito de Fumar, Política Pública, Personal de Salud

## Abstract

Em um país com cerca de 20 milhões de fumantes adultos, 174 mil mortes anuais e R$ 153,5 bilhões anuais entre custos diretos e indiretos atribuíveis ao tabagismo, a oferta de aconselhamento breve para parar de fumar pelo profissional de saúde durante qualquer visita de rotina, conforme preconizado pela Organização Mundial da Saúde (OMS), tem enorme potencial de redução desses números. Estimou-se para homens e mulheres com 35 anos ou mais, a partir dos dados nacionais mais recentes de comportamento de fumar e de custos de doenças selecionadas (doenças cardíacas isquêmicas, acidente vascular cerebral, doença pulmonar obstrutiva crônica e câncer de pulmão), as mortes e os custos totais que poderiam ter sido evitados caso o aconselhamento breve tivesse sido oferecido a todos os fumantes no Brasil em 2019. Se todos os 7,1 milhões de fumantes que não receberam aconselhamento breve (49,7% do total) tivessem sido abordados, teríamos tido meio milhão a mais de ex-fumantes. Essa cessação teria sido equivalente a uma redução de 642 mortes e uma economia, à época, de R$ 749,9 milhões para a sociedade. Os maiores números de mortes e custos evitados teriam sido observados, respectivamente, entre os homens para doenças cardíacas e entre as mulheres para acidente vascular cerebral. O Brasil tem ainda um elevado número absoluto de fumantes e, portanto, os altos custos associados ao tabagismo deveriam ser analisados sob a perspectiva de uma abordagem populacional focada na redução de novos casos e mortes por doenças relacionadas ao tabaco. Os recursos economizados poderiam, assim, ser direcionados para atender às necessidades básicas da população, promovendo a saúde e melhorando a qualidade de vida.

## Abstract (en)

In a country with about 20 million adult smokers, 174,000 deaths per year and BRL 153.5 billion in annual direct and indirect costs are attributable to smoking. The provision of brief advice to quit smoking by health professionals during any routine visit, as recommended by the World Health Organization, has enormous potential to reduce these numbers. For men and women aged 35 years or older, we estimated the deaths and total costs that could have been avoided if brief counseling had been offered to all smokers in Brazil in 2019 based on the most recent national data on smoking behavior and the costs of selected diseases (ischemic heart disease, stroke, chronic obstructive pulmonary disease, and lung cancer). If all 7.1 million smokers who did not receive brief counseling (49.7% of the total) had been approached, we could have had half a million more former smokers, i.e., representing a reduction of 642 deaths and saving, at the time, BRL 749.9 million. The highest numbers of deaths and cost savings would have been observed, respectively, among men for heart disease prevention and women for stroke prevention. Brazil still has a high absolute number of smokers and, therefore, the high costs associated with smoking should be analyzed using a population-based approach focused on reducing new cases and deaths from tobacco-related diseases. The resources saved by this strategy could be directed to meet the basic needs of the population, promoting health and improving quality of life.

## Abstract (es)

En un país con cerca de 20 millones de fumadores adultos, 174 mil muertes anuales y un costo de BRL 153,5 mil millones al año entre costes directos e indirectos atribuibles al tabaquismo, la oferta de asesoramiento breve para dejar de fumar por parte de los profesionales sanitarios durante cualquier visita rutinaria, tal y como recomienda la Organización Mundial de la Salud, tiene un enorme potencial para reducir estas cifras. A partir de los datos nacionales más recientes sobre comportamiento tabáquico y costos de enfermedades seleccionadas (enfermedad cardíaca isquémica, accidente cerebrovascular, enfermedad pulmonar obstructiva crónica y cáncer de pulmón), se estimaron las muertes y costos totales que podrían haberse evitado en 2019 si el asesoramiento breve se hubiera ofrecido a todos los fumadores en Brasil, hombres y mujeres de 35 años o más. Si se hubiera abordado a los 7,1 millones de fumadores que no recibieron asesoramiento breve (el 49,7% del total), habríamos tenido medio millón más de exfumadores. Esta disminución equivaldría a una reducción de 642 muertes y un ahorro, para la época, de BRL 749,9 millones para la sociedad. Las mayores cifras de muertes y costos evitados se observarían, respectivamente, entre los hombres por enfermedades cardíacas y las mujeres por accidentes cerebrovasculares. Brasil sigue teniendo un elevado número de fumadores y, por lo tanto, los altos costes asociados al tabaquismo deberían analizarse desde la perspectiva de un enfoque poblacional centrado en la reducción de nuevos casos y muertes por enfermedades relacionadas con el tabaco. Los recursos ahorrados podrían destinarse así a satisfacer las necesidades básicas de la población, promoviendo la salud y mejorando la calidad de vida.

## Introdução

Apesar de o Brasil ter reduzido consideravelmente a proporção de fumantes nas últimas décadas, a última estimativa nacional apontou que ainda existem cerca de 20 milhões de fumantes adultos ^1^. Sendo o uso de produtos derivados do tabaco uma das causas componentes de uma parcela significativa de causas suficientes para o desenvolvimento de doenças cardiovasculares, pulmonares e mais de 15 tipos de câncer ^2^, não por acaso o tabagismo ainda causa cerca de 174 mil mortes anuais no Brasil ^3^. Ademais, as doenças associadas a tal comportamento de risco representam cerca de R$ 153,5 bilhões anuais em custos diretos e indiretos para o país ^3^.

Em uma publicação recente da Organização Mundial da Saúde (OMS) referente às diretrizes para o tratamento clínico para a cessação do tabagismo ^4^, foi recomendado o aconselhamento breve, com duração de 30 segundos a 3 minutos durante qualquer visita de rotina a um profissional de saúde. Tal recomendação se justifica pelo enorme número de fumantes que podem ser acessados com o baixo custo envolvido ^4^.

Os dados mais atualizados do sistema de monitoramento da epidemia do tabagismo no Brasil apontaram que quase 50% dos fumantes que estiveram, em 2019, diante de um profissional de saúde por qualquer motivo de saúde não receberam aconselhamento breve, o que representa cerca de 10 milhões de indivíduos ^1^ ^,^ ^5^. Apesar de este aconselhamento não envolver altas taxas específicas de cessação a médio prazo quando comparado aos outros métodos cognitivo-comportamentais e/ou farmacológicos recomendados ^4^ ^,^ ^6^, o impacto populacional em termos de redução do número de fumantes e, consequentemente, dos custos de doenças relacionadas ao tabaco pode ser expressivo - principalmente para um país com dimensões continentais e escassez de recursos como o Brasil, mas que conseguiu expandir consideravelmente o acesso ao serviço de saúde da população nos últimos anos ^5^.

O objetivo desse artigo é, portanto, estimar, a partir dos dados nacionais mais recentes de comportamento de fumar e de custos das doenças relacionadas ao tabaco, os respectivos números de mortes e de custos que poderiam ter sido evitados caso o aconselhamento breve tivesse sido oferecido a todos os fumantes no Brasil em 2019.

## Métodos

Esse artigo utiliza os dados de prevalência de fumantes atuais de qualquer produto derivado do tabaco, estratificados por sexo, oriundos da *Pesquisa Nacional de Saúde* (PNS) conduzida em 2019 ^1^. Essa pesquisa foi realizada pelo Instituto Brasileiro de Geografia e Estatística (IBGE) com uso de uma amostra probabilística estratificada e ponderada, com quatro etapas de seleção (municípios, setores censitários, domicílios e indivíduos com 15 anos ou mais). Maiores detalhes sobre a metodologia podem ser encontrados em publicação específica ^1^.

As duas perguntas que definiram se o fumante recebeu ou não aconselhamento breve em qualquer visita de rotina a um profissional de saúde foram as seguintes: (1) “Nos últimos doze meses, durante algum atendimento por médico ou outro profissional de saúde, foi perguntado se o(a) Sr(a) fumava?”, sendo que as opções de resposta eram “Sim”, “Não” e “Não passou por atendimento com profissional de saúde nos últimos doze meses”; para aqueles que responderam afirmativamente, perguntou-se se (2) “Nos últimos doze meses, durante algum desses atendimentos, o(a) Sr(a) foi aconselhado(a) a parar de fumar?”, com opções de resposta “Sim” e “Não”. Aqueles que responderam negativamente às perguntas 1 e 2, incluindo os que não tiveram acesso a profissionais de saúde no último ano, foram classificados como não tendo recebido aconselhamento breve. Calculou-se, ainda, a proporção de fumantes atuais que estavam tentando parar de fumar segundo status de aconselhamento breve recebido, por meio da pergunta: “Durante os últimos doze meses, o(a) Sr(a) tentou parar de fumar?”. Finalmente, entre aqueles que estavam tentando parar de fumar, foi perguntado se estavam usando aconselhamento, incluindo o cognitivo-comportamental intensivo, e/ou farmacoterapia; a resposta negativa para ambas as opções indicava que o fumante estava tentando parar de fumar sozinho.

Os dados de custos associados ao uso de produtos derivados do tabaco são provenientes de um estudo de carga da doença e carga econômica atribuível ao tabagismo conduzido em 2022 no Brasil ^3^. O estudo estimou a mortalidade, a morbidade e os custos (direto da assistência médica, indireto por perda de produtividade por morte prematura, por incapacidade e por perda de produtividade do cuidador informal) atribuíveis ao tabagismo entre indivíduos com 35 anos ou mais de idade. Tais dados possibilitaram, ainda, calcular, para cada doença analisada, o custo total equivalente a uma morte. Maiores detalhes sobre os parâmetros do modelo matemático de microssimulação probabilística utilizados, envolvendo status de fumante, riscos de incidência e mortalidade dos desfechos selecionados e respectivos impactos econômicos, podem ser encontrados em relatório específico ^7^.

A análise contrafactual do número de mortes e respectivos custos totais (diretos e indiretos) que poderiam ter sido evitados caso o aconselhamento breve tivesse sido oferecido a todos os fumantes no Brasil em 2019 foi limitada à população de 35 anos ou mais de idade e às doenças cardíacas isquêmicas (códigos I20-I25 da 10ª revisão da Classificação Internacional de Doenças - CID-10), doença pulmonar obstrutiva crônica (CID-10 J40-J44), acidente vascular cerebral (CID-10 I60-I69) e câncer de pulmão (CID-10 C33-C34). A restrição das doenças selecionadas foi baseada na disponibilidade de utilizar as respectivas taxas de mortalidade brutas por sexo e faixa etária para fumantes, ex-fumantes e nunca fumantes, oriundas de um conjunto de cinco estudos de coorte conduzidos nos Estados Unidos ^2^ ^,^ ^8^ ^,^ ^9^. De posse dessas informações, pôde-se calcular, para ambos os sexos, os riscos relativos de mortalidade e os respectivos intervalos de 95% de confiança (fumantes *versus* nunca fumantes [RR_1_]; ex-fumantes *versus* nunca fumantes [RR_2_]) ajustados por idade para cada um dos quatro desfechos de saúde selecionados, utilizando como população padrão a população brasileira de 2019 ^8^ ^,^ ^10^.

O número de mortes atribuíveis ao tabagismo (MAT), a partir da fração atribuível (FAT) para as doenças selecionadas e do número de mortes totais provenientes do Sistema de Informações sobre Mortalidade (SIM) ^10^, foi estimado separadamente para homens e mulheres a partir das seguintes fórmulas ^11^:

$$FAT=\frac{p_{0}+p_{1}\times {RR}_{1}+p_{2}\times {RR}_{2}-1}{p_{0}+p_{1}\times {RR}_{1}+p_{2}\times {RR}_{2}}$$ (1)

onde *p*_*0*_ = proporção de nunca fumantes; *p*_*1*_ = proporção de fumantes atuais; e *p*_*2*_ = proporção de ex-fumantes.

MAT=FAT×Mortes totais (2)

Posteriormente, para calcular a FAT_contrafactual_ e o número de mortes e custos evitados, considerando que uma parcela de fumantes atuais teria recebido aconselhamento breve e se tornado, portanto, ex-fumante, foram levadas em consideração as seguintes informações oriundas da PNS ^1^ ^,^ ^5^: (i) proporções de não aconselhamento breve por sexo na população de 35 anos ou mais (*p*_*3*_ ) e (ii) as respectivas proporções de não tentativa de parar de fumar (*p*_*4*_ ), tentativa de parar de fumar sozinho (*p*_*5*_ ) ou com apoio (*p*_*6*_ ). Levou-se, ainda, em consideração, o (iii) excesso esperado nas taxas de cessação por pelo menos seis meses (ETC) entre quem teria recebido aconselhamento breve sem estar tentando previamente parar de fumar (ETC_1_ = 8,6%) ^4^ ^,^ ^6^, tentando parar de fumar sozinho (ETC_2_ = 1,5%) ^4^ ^,^ ^6^ ou tentando parar de fumar e já usando algum apoio (ETC_3_ = 0,0%) ^4^ ^,^ ^6^. Sendo assim, a nova fórmula da FAT seria:

$${FAT}_{contrafactual}=\frac{p_{0}+(p_{1}-p_{1contrafactual})\times {RR}_{1}+(p_{2}+p_{1contrafactual})\times {RR}_{2}-1}{p_{0}+{\left(p_{1}-p_{1contrafactual}\right)}_{1}\times {RR}_{1}+(p_{2}+p_{1contrafactual})\times {RR}_{2}}$$ (3)

onde *p*_*1contrafactual*_ *= p*_*1*_ *× p*_*3*_ *× p*_*4*_ *× ETC*_*1*_ *+ p*_*1*_ *× p*_*3*_ *× p*_*5*_ *× ETC*_*2*_ *+ p*_*1*_ *× p*_*3*_ *× p*_*6*_ *× ETC*_*3*_ .

E o número de mortes (ME) e custos evitados (CE), estes últimos corrigidos pelo ajuste inflacionário medido pela variação do Índice Nacional de Preços ao Consumidor Amplo (IPCA) entre 2019 e 2022 ^12^, por doença específica e sexo, foram derivados das seguintes fórmulas:

$$ME=\left(FAT-{FAT}_{contrafactual}\right)\times Mortes\;totais$$ (4)

CE=ME×Custo equivalente a 1 morte associada ao tabaco (5)

O artigo utilizou dados de domínio público, não identificados, sendo dispensada a submissão ao sistema CEP/CONEP (Comissão Nacional de Ética em Pesquisa) de acordo com a legislação em vigor.

## Resultados

Em 2019, 4,4 milhões de fumantes homens e 2,7 milhões de fumantes mulheres não receberam aconselhamento breve para parar de fumar em qualquer visita de rotina a um profissional de saúde. Para ambos os sexos, aqueles que não receberam aconselhamento breve tiveram uma maior proporção de fumantes que não tentaram parar de fumar, ou menor proporção de apoio medicamentoso (isolado ou combinado com aconselhamento), quando comparados aos que receberam aconselhamento breve. Se todos os fumantes tivessem recebido aconselhamento breve em 2019, teríamos tido meio milhão a mais de ex-fumantes (Tabela 1).

**Tabela 1:** Distribuição dos fumantes de 35 anos ou mais segundo *status* de aconselhamento breve recebido, *status* de tentativa de parar de fumar e número de ex-fumantes estimado a partir da análise contrafactual, estratificado por sexo *. Brasil, 2019.

Variáveis selecionadas	Homens		Mulheres	
	Aconselhamento breve	Não aconselhamento breve **	Aconselhamento breve	Não aconselhamento breve **
Número de fumantes (em milhões) - cenário 2019 ***	3,5	4,4	3,7	2,7
Tentativa de parar de fumar últimos 12 meses (%)				
Não	54,9	69,5 ^#^	48,1	58,9 ^#^
Sim, sozinho	31,1	28,0	33,3	34,6
Sim, apoio com aconselhamento apenas ^##^	6,5	0,7 ^#^	7,8	2,8 ^#^
Sim, apoio medicamentoso (isolado ou combinado com aconselhamento ^##^)	7,5	1,8 ^#^	10,8	3,7 ^#^
Número de fumantes (em milhões) a mais que teriam parado de fumar - cenário contrafactual 2019 ^###^	NA	0,3	NA	0,2

NA: não se aplica.

* Todos as análises foram realizadas considerando os pesos amostrais dos indivíduos selecionados pela *Pesquisa Nacional de Saúde* de 2019 ^1^;

** O não aconselhamento breve incluiu 15,2% do total de homens fumantes e 6,1% do total de mulheres fumantes que não visitaram um profissional de saúde no último ano;

*** Prevalências de fumantes atuais de 35 anos ou mais de idade ^1^: homens: 16% e mulheres: 10,8%;

^#^ Valor de p ≤ 0,05 na comparação das proporções de “não tentativa de parar de fumar” (ou de “tentativa de parar de fumar com apoio aconselhamento” ou de “tentativa de parar de fumar com apoio medicamentoso (isolado ou combinado com aconselhamento)”) entre quem recebeu aconselhamento breve e entre quem não recebeu aconselhamento breve, separadamente para homens e mulheres;

^##^ Pode incluir tanto o aconselhamento breve quanto aquele cognitivo-comportamental mais intensivo;

^###^ Considerou-se o excesso de fumantes a partir do cálculo a seguir: fumantes a mais que teriam virado ex-fumantes=número atual de fumantes não aconselhados×”% não tentativa de parar de fumar”×0,086 + número atual de fumantes não aconselhados×”% tentativa de parar de fumar sozinho”×0,015. As prevalências de ex-fumantes de 35 anos ou mais de idade do cenário de referência (ano 2019 ^1^) eram: homens: 31,9% e mulheres: 29%.

O impacto populacional que teria sido obtido em termos de mortes e custos totais evitados, caso o aconselhamento breve tivesse sido oferecido a todos os fumantes em uma visita de rotina em 2019, foi equivalente, respectivamente, a 642 mortes e R$ 749,9 milhões. Para ambos os sexos, o câncer de pulmão teria sido o desfecho com o menor impacto populacional, sendo que os maiores números de mortes e custos evitados teriam sido observados, respectivamente, entre os homens para doenças cardíacas e entre as mulheres para acidente vascular cerebral (Tabela 2).

Detalhes dos RRs e FATs utilizadas se encontram na Tabela S1 (Material Suplementar; https://cadernos.ensp.fiocruz.br/static//arquivo/suppl-e00000125_2852.pdf).

**Tabela 2:** Distribuição das mortes e custos atribuíveis ao tabagismo, segundo sexo e cenário de análise *. Indivíduos de 35 anos ou mais de idade, Brasil, 2019.

Doenças selecionadas **	Mortes atuais atribuíveis ao tabagismo	Custo equivalente a 1 morte (R$ × 10^3^) ***	Mortes atribuíveis ao tabagismo evitadas ^#^	Custo total evitado (R$ × 10^3^)
Câncer de pulmão ^##^				
Homens	13.634	370,5	44	16.302,0
Mulheres	9.533	360,0	32	11.520,0
Total	23.168	366,9	76	27.884,0
DPOC ^###^				
Homens	22.290	849,1	47	39.907,7
Mulheres	17.473	882,4	35	30.884,0
Total	39.763	862,1	82	70.692,2
AVC ^§^				
Homens	8.190	2064,1	120	247.692,0
Mulheres	5.597	2662,0	99	263.538,0
Total	13.787	2296,8	219	502.999,2
Doenças cardíacas isquêmicas ^§§^				
Homens	22.594	572,9	174	99.684,6
Mulheres	9.613	443,4	91	40.349,4
Total	32.207	539,2	265	140.034,0
Todas as doenças selecionadas ^§§§^				
Homens	66.708	NA	385	403.583,6
Mulheres	42.216	NA	257	346.291,1
Total	108.924	NA	642	749.874,7

AVC: acidente vascular cerebral; CID-10: Classificação Internacional de Doenças - 10ª revisão; DPOC: doença pulmonar obstrutiva crônica; IPCA: Índice Nacional de Preços ao Consumidor Amplo; NA: não se aplica.

* Considerando que uma parcela de fumantes atuais teria recebido aconselhamento breve e teria virado, portanto, ex-fumante. Os parâmetros utilizados podem ser encontrados na Tabela 1 e na Tabela S1 (Material Suplementar; https://cadernos.ensp.fiocruz.br/static//arquivo/suppl-e00000125_2852.pdf);

** Câncer de pulmão (CID-10 C33-C34), DPOC (CID-10 J40-J44), AVC (CID-10 I60-I69) e doenças cardíacas isquêmicas (CID-10 I20-I25);

*** A correção pelo ajuste inflacionário medido pela variação do IPCA ^12^ entre o “custo total (custos direto da assistência médica, indireto por perda de produtividade por morte prematura, por incapacidade e por perda de produtividade do cuidador informal atribuíveis ao tabagismo) equivalente a 1 morte” em 2022 ^3^ e 2019 (ano de referência da análise contrafactual) foi de: 1/1,221;

^#^ Número de eventos atribuíveis ao tabagismo (homens e mulheres) evitados baseado na relação entre “eventos por morte” ^3^: câncer de pulmão (54 e 40), DPOC (538 e 406), AVC (667 e 654) e doenças cardíacas isquêmicas (973 e 508);

^##^ Intervalo de 95% de confiança para, respectivamente, “Mortes atuais atribuíveis ao tabagismo”, “Mortes atribuíveis ao tabagismo evitadas” e “Custo total evitado” relacionados ao câncer de pulmão: homens: 13.175-16.333, 37-50, 13.708,5-18.525,0 e mulheres: 9.140-12.545, 29-36, 10.440-12.960.

^###^ Intervalo de 95% de confiança para, respectivamente, “Mortes atuais atribuíveis ao tabagismo”, “Mortes atribuíveis ao tabagismo evitadas” e “Custo total evitado” relacionados à DPOC: homens: 21.578-22.865, 35-57, 32.265,8-48.398,7 e mulheres: 6.755-18.092, 31-41, 27.354,4-31.178,4;

^§^ Intervalo de 95% de confiança para, respectivamente, “Mortes atuais atribuíveis ao tabagismo”, “Mortes atribuíveis ao tabagismo evitadas” e “Custo total evitado” relacionados ao AVC: homens: 3.795-11.824, 119-123, 245.627,9-253.884,3 e mulheres: 2.180-7.090, 88-107, 234.256,0-284.834,0;

^§§^ Intervalo de 95% de confiança para, respectivamente, “Mortes atuais atribuíveis ao tabagismo”, “Mortes atribuíveis ao tabagismo evitadas” e “Custo total evitado” relacionados às doenças cardíacas isquêmicas: homens: 20.505-24.389, 169-175, 96.820,1-100.257,5 e mulheres: 7.223-11.594, 91-93, 40.349,4-41.236,2;

^§§§^ Intervalo de 95% de confiança para, respectivamente, “Mortes atuais atribuíveis ao tabagismo”, “Mortes atribuíveis ao tabagismo evitadas” e “Custo total evitado” relacionados ao total das doenças selecionadas: homens: 59.053-75.411, 363-405, 388.422,3-421.065,5 e mulheres: 35.298-49.321, 239-277, 312.399,8-370.208,6.

## Discussão

A estratégia populacional de fornecer aconselhamento breve a todos os fumantes brasileiros durante visitas de rotina pode se reverter em enormes ganhos de mortes e custos evitados. De fato, medidas de saúde pública antitabagismo que impactam a população como um todo, tais como as advertências sanitárias nas embalagens, a proteção contra o fumo passivo em locais públicos fechados e o aumento de preços e impostos ^13^, mostram-se mais custo-efetivas em termos de prevenção e controle das doenças associadas ao tabaco do que as estratégias baseadas em subgrupos de indivíduos de alto risco ^4^ ^,^ ^14^.

A expansão do acesso aos serviços de saúde em todas as regiões do Brasil ^15^ reforça a importância dos achados deste estudo, haja vista que o aconselhamento breve não envolve efeitos adversos do uso de medicamentos, é de baixo custo e apresenta poucas dificuldades de aceitação pela sociedade ^4^. Para um país onde a proporção de fumantes está concentrada nos subgrupos de menor renda e escolaridade ^1^, o aconselhamento breve pode ter, portanto, um potencial impacto positivo na redução da iniquidade na distribuição de usuários de produtos derivados do tabaco no Brasil ^1^ ^,^ ^4^. No entanto, realidades econômicas, políticas, físicas e culturais distintas entre as regiões brasileiras (e.g., distância, tempo de espera e/ou dificuldade de marcação de consultas) podem reduzir o impacto populacional esperado ^1^.

Esses achados podem ser úteis, ainda, para uma reflexão entre os países sobre os desafios de se obter a efetiva implementação da recomendação da OMS ^4^. Embora o Brasil apresente uma proporção de fumantes que não receberam aconselhamento breve de cerca de 50%, estudos recentes realizados, inclusive em países desenvolvidos como os Estados Unidos e os Países Baixos, encontraram proporções significativamente maiores de fumantes que não receberam aconselhamento para parar de fumar durante consultas na atenção primária (77,9% e 67,1%, respectivamente) ^16^ ^,^ ^17^.

Nesse sentido, é fundamental que os profissionais de saúde, tanto os atuais quanto os futuros que ainda estão se formando nas universidades, tenham conhecimento técnico e apoio de estrutura e recursos para serem efetivamente modelos de comportamento para o paciente e/ou a sociedade ^5^. O aconselhamento breve pode ajudar todos os usuários de produtos derivados de tabaco, independentemente do interesse em parar de fumar. E esse aconselhamento breve em todos os níveis de atenção à saúde serve como um apoio adicional à cessação (e.g., tratamento cognitivo-comportamental, com ou sem uso de medicação). Trata-se de uma estratégia que vai ao encontro da implementação da Convenção-Quadro sobre Controle do Uso do Tabaco ^13^, ratificada pelo Brasil há 20 anos; e, em particular, do seu Art. 14, que reforça que todos os países devem implementar programas para o diagnóstico, aconselhamento, prevenção e tratamento da dependência do tabaco nos serviços de saúde, com a participação de profissionais de saúde.

A *Portaria GM/MS nº 502/2023*, que instituiu o Programa Nacional de Controle do Tabagismo (PNCT), criado em 1989, no âmbito do Sistema Único de Saúde (SUS), fortalece o aconselhamento breve durante visitas de rotina ao indicar, como um dos seus eixos estruturantes, o cuidado integral, incluindo ações de prevenção e promoção da saúde ^18^. Cabe ressaltar, ainda, que o PNCT, no ano 2004, ampliou o acesso à abordagem e ao tratamento do tabagismo para a rede de atenção básica no SUS ^18^.

A grande maioria dos fumantes ainda não está tentando parar de fumar e/ou está tentando parar de fumar sozinha ^1^ ^,^ ^5^. Paralelamente à oferta de aconselhamento breve a todos os fumantes e/ou de tratamento estruturado na rede de saúde, é fundamental, portanto, que o país continue avançando na efetiva implementação de outras medidas direcionadas à prevenção da iniciação e ao estímulo da cessação ^13^ ^,^ ^19^. Apesar da queda observada nos últimos anos na proporção de fumantes no Brasil ^1^, as ações de interferência da indústria do tabaco para recrutar novos fumantes e/ou impedir que os fumantes atuais se libertem da dependência da nicotina - tais como o marketing agressivo em prol dos dispositivos eletrônicos para fumar e/ou ações judiciais que bloqueiam a proibição de produtos de tabaco com aromas e sabores no Brasil ^19^ - reforçam a importância da sinergia almejada entre as medidas antitabagismo presentes e futuras. Nesse sentido, a reforma tributária em discussão no Congresso Nacional constitui uma oportunidade singular para elevar novamente a carga tributária sobre os cigarros industrializados e, com isso, diminuir o número de seus consumidores.

Ao se utilizarem os dados de custos diretos e indiretos das doenças relacionadas ao tabaco selecionadas de 2022 ^4^ para a análise contrafactual realizada tendo 2019 como ano de referência, assumiu-se que, entre estes anos, o país teve uma população aberta e estável para as doenças analisadas (prevalência constante), mantendo-se, portanto, inalterada a relação entre mortes evitadas e casos novos evitados. O impacto populacional da análise contrafactual apresentada está provavelmente subestimado, haja vista que (i) não foram considerados os custos envolvendo outras doenças, tais como diabetes e outros 14 tipos de câncer ^2^ ^,^ ^3^, e (ii) as taxas de cessação envolvendo os fumantes que teriam recebido aconselhamento breve poderiam ter sido ainda maiores em função do aumento de busca adicional por apoio cognitivo-comportamental intensivo e/ou medicamentoso oferecido gratuitamente no país ^4^ ^,^ ^5^ ^,^ ^6^.

## Conclusão

Em um país com um elevado número absoluto de fumantes, os altos custos associados ao consumo de produtos derivados do tabaco deveriam ser analisados sob a perspectiva de uma abordagem populacional focada na redução de novos casos e mortes por doenças relacionadas ao tabaco. Para tal, o aconselhamento breve recomendado pela OMS seria uma opção custo-efetiva. Os recursos economizados poderiam ser, assim, direcionados para atender às necessidades básicas da população, promovendo a saúde e melhorando a qualidade de vida.
