# Supplementary material for: Brief counseling in routine consultations: a population-based strategy to reduce the disease and the economic burdens of smoking in Brazil
Source: Cad Saude Publica. 2025 Aug 8;41(7):e00000125. [Article in Portuguese] doi: 10.1590/0102-311XPT000125 (PMC12334165; doi:10.1590/0102-311XPT000125)
Supplement: Supplementary file 1 [file 1678-4464-csp-41-07-PT000125-s.pdf]

MATERIAL SUPLEMENTAR

Tabela S1 Distribuição dos riscos relativos ajustados pela idade[1,2] e das frações atribuíveis ao tabagismo para doenças selecionadas, segundo sexo e cenário de análise[3]. Indivíduos de 35 anos ou mais de idade. Brasil, 2019.

Doenças selecionadas[4]	Homens				Mulheres			
	Fumantes RR[5,6]	Ex-fumantes RR[5,6]	FAT (%)[7]	FAT Contrafactual (%)[7]	Fumantes RR[5,6]	Ex-fumantes RR[5,6]	FAT (%)[7]	FAT Contrafactual (%)[7]
Câncer de pulmão	22,8	5,9	83,5	83,3	19,6	5,0	76,0	75,8
DPOC	32,2	9,0	88,3	88,1	23,4)	7,7	81,4	81,2
AVC	1,8	1,2	16,2	15,9	2,0	1,1	11,4	11,2
Doenças cardíacas isquêmicas	2,7	1,7	33,3	33,0	2,3	1,4	20,0	19,8

Notas:

RR = Risco Relativo; FAT = Frações atribuíveis ao tabagismo; DPOC=Doença Pulmonar Obstrutiva Crônica; AVC=Acidente Vascular Cerebral

[1] A população de referência utilizada para o ajuste direto por idade foi a população brasileira de 2019 (referência #10 do artigo). As distribuições por idade consideradas foram: 35-39 (17,7%), 40-44 (15,7%), 45-49 (13,8%), 50-54 (12,7%), 55-64 (20,5%), 65-74 (12,2%) e 75+ (7,4%).

[2] As taxas brutas de mortalidade por faixa etária para as doenças selecionadas para fumantes, ex-fumantes e nunca fumantes, assim como a metodologia de cálculo utilizada, se encontram disponíveis nas referências #8 e #9 do artigo.

[3] Considerando que uma parcela de fumantes atuais teria recebido aconselhamento breve e teria virado, portanto, ex-fumante. Foram levadas em consideração as informações oriundas da Pesquisa Nacional de Saúde de 2019 (vide referência #1 do artigo) e contidas na tabela 1 do artigo, além do excesso nas taxas de cessação (ETC) por 6 meses ou mais esperadas entre quem teria recebido aconselhamento breve sem estar tentando previamente de parar de fumar (ETC1=8,6%) ou tentando parar de fumar sozinho (ETC2=1,5%).

[4] Câncer de pulmão (CID-10: C33-C34), Doença Pulmonar Obstrutiva Crônica (DPOC) (CID-10: J40–J44), Acidente Vascular cerebral (AVC) (CID-10: I60–I69) e Doenças cardíacas isquêmicas (CID-10: I20–I25).

[5] Referência: nunca fumantes

[6] Os intervalos de confiança de 95% das razões de taxas ajustadas pela idade foram calculados a partir da fórmula descrita em "Boyle P, Parkin DM. Cancer registration: principles and methods. Statistical methods for registries. IARC Sci Publ 1991;(95):126-58". Homens, RR fumantes e RR ex-fumantes: Câncer de pulmão (19,1-27,1) e (5,0-7,1); DPOC (25,6-40,5) e (7,1-11,3); AVC (15,-2,1) e (1,0-1,4); Doenças cardíacas isquêmicas (2,5-2,9) e (1,6-1,8). Mulheres, RR fumantes e RR ex-fumantes: Câncer de pulmão (17,0-22,5) e (4,3-5,7); DPOC (19,4-28,3) e (6,4-9,3); AVC (1,7-2,3) e (1,0-1,2); Doenças cardíacas isquêmicas (2,1-2,6) e (1,2-1,5).

[7] Intervalos de confiança de 95% (cenário atual e contrafactual) para os homens: Câncer de Pulmão (80,7-86,0) e (80,4-85,7); DPOC (85,4-90,6) e (85,2-90,4), AVC (7,5-23,3) e (4,5-14,5), Doenças cardíacas isquêmicas (30,2-35,9) e (29,9-35,6). Para as mulheres: Câncer de Pulmão (72,9-78,7) e (72,6-78,4); DPOC (78,0-84,3) e (77,8-84,1), AVC (7,2 -23,1) e (4,2-14,2), Doenças cardíacas isquêmicas (15,0-24,1) e (14,8-23,9).
